# The Potential Role of Quorum Sensing in Rumen Microbial Adaptation to Environmental and Nutritional Stress: A Review

**DOI:** 10.3390/ani16152356

**Published:** 2026-08-02

**Authors:** Chang Liu, Kehui Ouyang, Mingren Qu, Qinghua Qiu

**Affiliations:** Jiangxi Province Key Laboratory of Animal Nutrition and Feed, College of Animal Science and Technology, Jiangxi Agricultural University, Nanchang 330045, China

**Keywords:** biofilm formation, fermentation characteristics, heat stress, nutritional stress, quorum sensing, rumen microbiota, stress mitigation, stress response

## Abstract

Stress-induced perturbations in rumen microbial community structure and function disrupt fermentation homeostasis, consequently impairing production performance and increasing health risks in ruminants. Quorum sensing (QS), a crucial mechanism governing microbial collective behavior and intercellular communication, is increasingly recognized for its role in ecological adaptation of rumen microbiota, yet direct evidence for its regulatory role in the rumen remains limited. This review summarizes the impacts of diverse stressors, including heat stress, cold stress, transport stress, and nutritional stress, on rumen fermentation characteristics and microbial community composition. Building upon this foundation, we discuss the alterations in QS signaling molecules and elucidate underlying microbial adaptation mechanisms from the perspectives of homeostasis disruption, signal transduction, and collective behavior regulation. Although QS-based nutritional and management strategies have shown preliminary potential for alleviating rumen stress, their effectiveness under practical production conditions requires further validation. This review provides a conceptual framework for understanding the potential role of QS in rumen microbial adaptation to environmental and nutritional stress, while highlighting current knowledge gaps and future research directions.

## Simple Summary

Stress caused by heat, cold, transport, or dietary changes can disrupt rumen microbial homeostasis, reducing productivity and increasing health risks in ruminants. Rumen microorganisms may utilize quorum sensing (QS), a communication system that coordinates collective microbial behaviors and facilitates adaptation to environmental changes. However, direct evidence linking QS signaling to microbial adaptation in the rumen, particularly under environmental stress conditions, remains scarce. This review summarizes current knowledge on how different stressors affect rumen fermentation, microbial communities, and QS-related signaling molecules, and discusses the potential involvement of QS in microbial adaptation. Understanding microbial communication may provide new insights into strategies for enhancing animal health and production efficiency.

## 1. Introduction

The rumen of ruminants is a highly complex anaerobic fermentation system inhabited by a diverse microbial community [1]. This microbial community forms a relatively stable ecosystem that facilitates cellulose degradation, volatile fatty acid (VFA) production, and microbial protein synthesis [2]. Maintenance of ruminal homeostasis is critical for animal growth performance, immune competence, and resilience to stress [3]. Stress refers to a series of physiological and behavioral responses that occur when animals encounter internal or external environmental changes [4,5,6,7]. Under intensive production systems, ruminants are continuously exposed to various environmental stressors, including cold stress, heat stress, transport stress, weaning stress, and abrupt dietary changes. These stressors can impair metabolic and immune functions, ultimately reducing production efficiency, with these effects potentially amplified through social transmission among group members [8]. Emerging evidence indicates that stress not only directly affects the host neuroendocrine system but also profoundly alters the rumen microbial environment by modifying pH, osmotic pressure, substrate availability, and redox balance [9,10,11,12].

To cope with fluctuating conditions, microorganisms have evolved a variety of adaptive strategies. Among these, quorum sensing (QS) is a widespread cell-to-cell communication mechanism that allows microbes to coordinate their behavior in response to population density [13,14]. As microbial populations increase, signaling molecules progressively accumulate in the extracellular environment. Upon reaching a threshold concentration, these signals are detected by neighboring cells and activate regulatory networks that synchronize gene expression and community-level functions such as biofilm formation, antibiotic production, luminescence, and sporulation [15,16,17]. The primary QS signaling molecules include acyl-homoserine lactones (AHL), autoinducer peptides (AIP), and autoinducer-2 (AI-2) [18,19]. AI-2 activity has been detected in a wide range of bacteria and has also been observed in rumen contents and monospecies cultures of rumen bacteria such as *Butyrivibrio fibrisolvens*, *Eubacterium ruminantium*, *Ruminococcus flavefaciens*, and *Succinimonas amylolytica,* suggesting that AI-2-based signaling occurs among rumen microorganisms [19,20,21,22]. However, systematic studies on the role of microbial QS in ruminal stress responses remain limited.

This review discusses the roles of QS in the adaptive responses of rumen microbiota to stress. By linking stress-induced changes in the ruminal environment to microbial signaling and coordinated behaviors, it provides a conceptual framework for understanding the mechanisms that maintain rumen homeostasis, which may guide the development of microbiota-targeted strategies to mitigate stress and enhance ruminant health and productivity.

## 2. Review Methodology

This review was conducted as a narrative synthesis aimed at integrating current evidence on QS-mediated microbial adaptation in the rumen under environmental and nutritional stress, with particular emphasis on microbial communication systems, stress-induced alterations in the rumen ecosystem, and the potential application of QS-targeted intervention strategies. Although narrative in structure, the review incorporated a structured literature search to enhance methodological transparency and reproducibility. Literature retrieval was performed in the Web of Science Core Collection and PubMed databases, covering English peer-reviewed articles from database inception to the final search date: 31 May 2026. Searches were limited to the title, abstract, and keyword fields, using the Boolean operators AND and OR to combine search terms. The full search string was as follows: (“rumen” OR “ruminant” OR “rumen microbiota” OR “rumen fermentation”) AND (“stress” OR “stress response” OR “environmental stress” OR “nutritional stress” OR “quorum sensing” OR “autoinducer-2” OR “LuxS” OR “N-acyl-homoserine lactone” OR “biofilm” OR “quorum sensing inhibitor”).

Additional relevant publications were identified through manual screening of reference lists from selected articles. Records were screened based on titles, abstracts, and full-text content for relevance to the scope of this review. Studies were included if they addressed one or more of the following aspects: (i) rumen microbial ecology and fermentation function; (ii) QS signaling and microbial communication; (iii) microbial stress adaptation; (iv) QS regulatory strategies for rumen microbes. Papers were excluded if they lacked relevance to the core themes, did not provide interpretable information related to microbial communication or stress adaptation, or lacked sufficient methodological details. Given the scarcity of rumen-specific QS research, mechanistic findings from non-ruminal microbes with conserved QS pathways were also incorporated.

**Figure 1:**
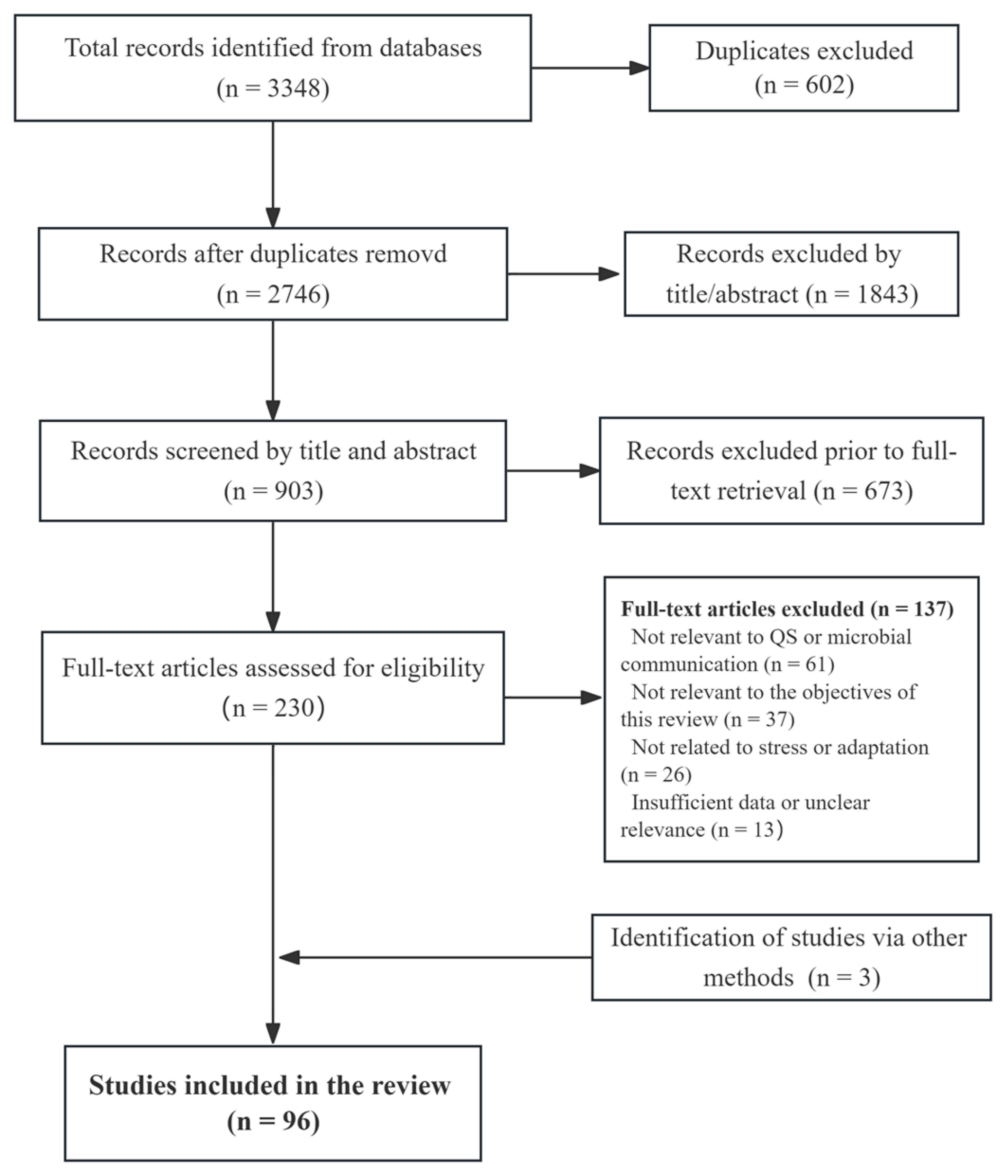
Flow diagram of the literature search and screening process.

The literature screening process was conducted independently by two reviewers. Any uncertainties or disagreements regarding the relevance of individual studies were resolved through discussion among the authors until consensus was reached. All included studies were qualitatively assessed across six predefined dimensions: experimental design type, in vitro or in vivo rumen model, ruminant species, sample size, detection methods for QS signal molecules, and alignment of experimental treatments with environmental or nutritional stress conditions, to judge methodological robustness and alignment with the review’s core theme. Due to substantial heterogeneity of study designs, microbial species, experimental models, analytical methodologies, and reported outcomes, a quantitative meta-analysis was not considered appropriate. The gathered evidence was organized into cohesive thematic sections according to stress type, QS mechanism, microbial adaptive response, and potential applications for rumen health and production. The full search and screening process is summarized in a flow diagram (Figure 1).

As this study was based exclusively on previously published material, no ethical approval was required.

## 3. Effects of Different Types of Stress on Rumen Fermentation and Microbial Communities

### 3.1. Heat Stress

Heat stress occurs when ambient temperature exceeds the upper limit of the animal’s thermoneutral zone [23]. Under high-temperature conditions, ruminants maintain thermal balance by reducing feed intake, increasing respiratory rate, and decreasing metabolic heat production [24]. Reduced feed intake and rumination activity lead to reduced salivary secretion. Because bicarbonate and phosphate in saliva are important buffering agents that maintain rumen pH stability, decreased salivary secretion weakens ruminal buffering capacity and causes a decline in rumen pH [23,25].

Heat stress is often associated with shifts in the rumen microbial community [26,27]. Studies have shown that heat stress in dairy cows increases the relative abundance of lactate-producing and starch-degrading bacteria while reducing the proportion of fiber-degrading bacteria in the rumen [28]. In Hanwoo steers subjected to acute heat stress, the relative abundance of fibrolytic Ruminococcaceae decreased, whereas that of lactate-producing Lactobacillaceae and amylolytic *Prevotella* and *Ruminobacter* increased [29]. A similar trend has been reported in goats. Heat stress reduced the abundance of beneficial members of the *Lachnospiraceae ND3007 group* and increased the abundance of potential pathogenic bacteria, such as *Erysipelotrichaceae UCG-004* and *Treponema 2* [30]. These microbial shifts may alter ruminal fermentation patterns from fiber degradation toward starch utilization, thereby reducing fiber utilization efficiency.

### 3.2. Cold Stress

In contrast to heat stress, cold stress typically occurs when the ambient temperature falls below the lower limit of the thermoneutral zone for ruminants. Under low-temperature conditions, ruminants increase heat production to maintain thermal balance, resulting in elevated energy requirements and increased feed intake [31]. However, this increased feed intake is often accompanied by an accelerated ruminal passage rate, which reduces feed retention time in the rumen and limits microbial attachment and degradation efficiency [32].

Cold exposure may directly decrease ruminal temperature, thereby influencing microbial fermentation activity. Both in vivo and in vitro studies have shown that cold water intake or exposure to low ambient temperatures can cause a rapid decrease in rumen temperature, which transiently inhibits microbial activity, fiber degradation, and overall fermentation rate [33,34]. Cold stress also shifts the production patterns of VFA in the rumen [35]. For instance, the proportion of propionate decreased, whereas acetate increased in sheep exposed to cold environments [36]. In Tibetan sheep, the concentrations of acetate, propionate, and butyrate are significantly higher in the cold season than in the warm season [37]. However, in grazing or extensive production systems, such seasonal differences may not solely reflect temperature-related stress, as cold seasons are often accompanied by changes in forage availability, dietary composition, and nutrient supply, which can also influence rumen fermentation and microbial community structure. Guo et al. [38] demonstrated that the cold season was associated with increased ruminal microbial diversity and changes in the relative abundance of fibrolytic bacteria in Tibetan sheep, although the specific contributions of temperature and seasonal nutritional factors require further investigation.

### 3.3. Transport Stress

Transport is an unavoidable management stressor in ruminant production and is often accompanied by fasting, dehydration, vibration, and overcrowding, all of which can induce stress responses [39,40]. Transport stress has been shown to activate the hypothalamus–pituitary–adrenal axis, resulting in increased secretion of stress hormones such as cortisol, as well as systemic immune and metabolic changes, and ultimately leading to disruption of physiological homeostasis [41,42].

Transport stress also affects rumen function. Long-distance transport alters ruminal fermentation characteristics, characterized by increased acetate concentrations, decreased propionate and butyrate concentrations, and a decline in ruminal pH [43]. These alterations are closely accompanied by dynamic shifts in the microbial community structure. Following transport stress, the cellulolytic bacteria *Fibrobacter succinogenes* and *Ruminococcus flavefaciens* transiently increased at 6 h post-transport and subsequently returned to baseline levels, whereas several carbohydrate-utilizing bacteria, including *Succinivibrio dextrinosolvens*, *Prevotella bryantii*, *Prevotella ruminicola*, and *Anaerovibrio lipolytica*, showed decreased abundance [43]. High-throughput sequencing studies further suggest that transport stress alters the relative abundances of dominant bacterial phyla, raising Firmicutes abundance while lowering Bacteroidetes levels [44]. In addition, transport stress can trigger systemic inflammatory responses. The concentrations of lipopolysaccharides in both serum and rumen fluid increase significantly following transport, accompanied by elevated expression of pro-inflammatory cytokines, interleukin-6, tumor necrosis factor-alpha, and interleukin-1 beta [44,45]. These inflammatory responses may further modify the ruminal metabolic environment and indirectly influence microbial community dynamics.

### 3.4. Nutritional Stress

Nutritional stress refers to metabolic disturbances caused by insufficient feed supply, abrupt dietary changes, and nutrient imbalance. This type of stress can affect ruminal fermentation characteristics and microbial community composition by altering substrate availability, ruminal pH, and microbial metabolic requirements [46]. Based on their origin, nutritional stressors can be broadly classified into two categories: physiological developmental transitions, represented by weaning, and management- or feed-associated nutritional stressors, including high-concentrate diets, substrate deprivation, mineral imbalances, variations in dietary nutrient composition (e.g., protein, fat, and organic matter concentrations), inappropriate use or dosage of feed additives, and mycotoxin contamination.

Weaning represents a physiological developmental transition during early life rather than a management-induced nutritional stressor. During this period, calves shift from a liquid-based diet to solid feed, resulting in substantial changes in ruminal fermentation substrates and driving rapid restructuring of the rumen microbial community [47]. Research has shown that the abundance of fiber-degrading bacteria decreases, whereas the proportions of starch-degrading and lactate-producing bacteria increase, ultimately leading to a reduction in ruminal pH and alterations in VFA profiles [48]. Such microbial shifts constitute an adaptive physiological response that facilitates rumen development and gastrointestinal maturation.

In contrast, other nutritional stressors generally arise from inappropriate feeding practices or dietary imbalances and are more likely to disrupt ruminal homeostasis. A primary example is the abrupt alteration of the forage-to-concentrate ratio. When the proportion of concentrate is excessively high, rapid ruminal starch fermentation produces large amounts of lactic acid, resulting in a marked decline in ruminal pH and potentially inducing subacute ruminal acidosis [49]. This environment promotes the proliferation of lactic acid-producing bacteria such as *Lactobacillus* and *Streptococcus bovis* while inhibiting the growth and metabolic activity of fiber-degrading microorganisms [50]. Conversely, insufficient substrate supply caused by severe feed restriction limits the availability of carbon and nitrogen sources required for microbial proliferation, thereby reducing microbial fermentation activity, total VFA production, and the abundance of saccharide-degrading bacteria, including *Saccharofermentans* and *Ruminococcus* [51]. In addition to disturbances in substrate availability, trace mineral imbalances caused by inappropriate diet formulation can also impair microbial metabolism [52]. For example, appropriate selenium supplementation significantly increases the abundance of fibrolytic bacteria, including *Ruminococcus albus*, *Ruminococcus flavefaciens*, and *Fibrobacter succinogenes*, thereby enhancing fiber degradation efficiency and VFA production [53]. Furthermore, mycotoxin-contaminated feed, particularly aflatoxin B1, represents another management-induced nutritional stressor that suppresses the secretion of fibrolytic enzymes by rumen microorganisms, disrupts VFA synthesis, and ultimately destabilizes ruminal fermentation homeostasis [54].

Despite distinct initiating factors and underlying mechanisms, different stressors commonly reshape the rumen ecosystem by altering substrate availability, ruminal pH, and metabolite profiles, thereby disrupting microbial community structure and fermentation function. Current research has primarily focused on stress-induced changes in ruminal fermentation characteristics and microbial composition. However, the regulatory mechanisms through which microorganisms coordinate population-level responses and achieve functional reorganization under stress conditions remain poorly understood and require further investigation.

## 4. Potential Regulatory Roles of Rumen Microbial QS in Stress Responses

In complex microbial ecosystems, community-level responses to environmental stress cannot be fully explained by individual metabolic adjustments alone. Increasing evidence suggests that microorganisms actively coordinate collective behaviors through QS, enabling populations to synchronize gene expression and potentially enhance adaptation to changing environmental conditions [55,56].

### 4.1. Effects of Stress on Rumen QS Signaling Molecules

As established in previous sections, diverse stressors can alter the ruminal microenvironment and microbial community structure [57,58]. These environmental fluctuations may influence QS signaling processes by affecting the production, stability, or accumulation of signaling molecules. The LuxS/AI-2 signaling system has been identified in gut and ruminal microorganisms [59,60,61], and environmental stressors may influence intercellular signaling and coordinated microbial behaviors of rumen microorganisms by modulating this system.

Under different stress conditions, the response characteristics of QS signaling molecules vary considerably. Regarding physical and chemical environmental changes, AHL signaling molecules are highly sensitive to pH. Yates et al. [62] found that, during the growth of *Yersinia pseudotuberculosis* and *Pseudomonas aeruginosa*, AHL is prone to lactone ring opening and inactivation under alkaline conditions but exhibits higher stability in relatively acidic environments. These chemical properties suggest that pH fluctuations associated with ruminal disturbances may influence the persistence and availability of AHL signals, thereby potentially affecting QS-mediated microbial communication.

Many studies have investigated changes in QS signaling molecules under nutritional stress. Decreasing the dietary concentrate-to-forage ratio (from 75:25 to 49:51) has been shown to increase ruminal microbial density and AI-2 concentrations, enhance biofilm formation, and upregulate the expression of the *ftsH* gene in *Prevotella* spp. [63]. In vitro fermentation studies further demonstrated that exogenous stressors, such as mycotoxins, can disrupt QS signaling and impair ruminal fermentation function. For example, zearalenone reduced AI-2 concentrations and inhibited the production of AHL signaling molecules, including C4-HSL, resulting in decreased VFA production and impaired fiber degradation [64]. Similarly, aflatoxin B1 decreased the concentrations of multiple QS signaling molecules, including AI-2, C4-HSL, C6-HSL, and 3-oxo-C6-HSL [65]. These findings suggest that QS-associated signaling may be involved in rumen microbial responses to nutritional stress by potentially influencing intercellular communication and metabolic cooperation.

Compared with studies on nutritional stress, investigations of QS under heat or cold stress conditions remain limited. Nevertheless, available evidence suggests that these stressors may indirectly influence QS activity by altering the ruminal microenvironment and microbial community structure. For example, upon alleviation of heat stress, significant shifts in ruminal microbial composition and metabolic profiles were observed, while QS signaling molecule concentrations remained relatively stable [66]. This uncoupling between microbial community shifts and stable QS signal concentrations suggests that the contribution of QS to microbial adaptation under heat stress remains largely hypothetical and requires further investigation. In contrast, increasing drinking water temperature under cold-stress conditions significantly elevates AI-2 concentrations and enhances biofilm formation while reducing oxidative stress [67]. Other stressors, such as transport, also disturb rumen microbial homeostasis; however, direct evidence linking transport stress to QS signaling remains unavailable.

Collectively, these studies indicate that stress-induced alterations in ruminal physicochemical conditions and microbial community composition are frequently accompanied by changes in QS signaling. QS dynamics are likely shaped by multiple interacting factors, including environmental conditions, signal stability, and microbial community composition, rather than by any single stressor alone. Therefore, the contribution of QS to rumen microbial stress adaptation should be considered context-dependent. Further studies are required to clarify the underlying regulatory mechanisms under diverse physiological and environmental conditions. A summary of the current evidence regarding the effects of different stressors on the ruminal environment, microbial communities, and QS signaling is provided in Table 1. To address the technical and experimental context of these findings, a comprehensive summary of the specific analytical methods, experimental models, applied stressors, and measured QS molecules across key studies is provided in Table 2.

### 4.2. QS-Mediated Microbial Stress Adaptation Mechanisms

Under stress conditions, QS systems have been suggested to contribute to microbial adaptation by orchestrating behaviors such as biofilm formation, antioxidant defense, and metabolic regulation [68,69,70,71]. However, direct mechanistic evidence within the rumen ecosystem remains limited. Most current insights derive from non-ruminal model bacteria, providing a comparative framework for generating hypotheses on how rumen microorganisms may utilize QS-mediated communication to respond to environmental stress.

Among the QS-regulated behaviors identified in model microorganisms, biofilm formation represents one of the best-characterized mechanisms associated with microbial persistence under environmental disturbances. For example, in *Pseudomonas aeruginosa*, the Las/Rhl system promotes biofilm development through coordinated regulation of *rhlAB* and *lasB* expression [68]. In *Vibrio cholerae*, the LuxO–HapR regulatory pathway controls biofilm formation and dispersal in a cell-density-dependent manner, thereby facilitating adaptation to environmental fluctuations [69,70]. Similarly, the LuxS/AI-2 system of *Lactiplantibacillus plantarum* enhances biofilm formation by promoting exopolysaccharide synthesis through LsrR-associated regulatory pathways [71]. Considering the complexity of the rumen ecosystem, the ecological significance of QS-regulated biofilm formation may vary among different microbial niches. Ruminal microorganisms are distributed across feed-particle-associated, liquid-associated, and epithelium-associated habitats, where biofilm formation may fulfill distinct functions. For example, QS-mediated extracellular polymeric substance production may potentially facilitate substrate attachment and microbial cooperation in feed-particle-associated communities, whereas in liquid- and epithelium-associated communities, QS may be more related to microbial communication, persistence, and host–microbe interactions.

Although the rumen is predominantly anaerobic, oxidative challenges may still occur at the rumen epithelial interface under specific conditions, such as epithelial inflammation and subacute ruminal acidosis [72]. These conditions can alter the rumen microenvironment and host–microbe interactions, potentially affecting the stress adaptation of epithelium-associated microorganisms [73]. In addition to biofilm regulation, QS has been implicated in microbial defense against oxidative and environmental stressors in several model bacterial systems, providing a theoretical basis for considering how QS-associated processes may contribute to stress responses in rumen-associated microbial communities. In *Pseudomonas aeruginosa*, QS activates antioxidant genes such as *katA* and *sodA*, thereby enhancing reactive oxygen species scavenging capacity and improving bacterial survival under oxidative stress conditions [74]. Likewise, the Com and LuxS/AI-2 systems in *Streptococcus* spp. participate in oxidative-stress and acid-stress responses by regulating DNA repair and other protective mechanisms, thereby increasing stress tolerance and environmental adaptability [75,76]. Beyond direct stress defense mechanisms, QS can also regulate broader survival strategies under nutrient limitation. In *Bacillus subtilis*, the ComQXPA system regulates the Spo0A signaling pathway to coordinate biofilm formation, sporulation, and dormancy, enabling long-term survival under nutrient-limited conditions [77,78].

However, these adaptive functions do not necessarily indicate that QS activation always produces beneficial ecological outcomes. In certain microbial populations, QS can promote cooperative behaviors, yet under different ecological settings, it may also regulate competitive interactions or alternative survival strategies [55,56]. For example, in the pathogenic bacterium *Staphylococcus aureus*, the *agr* system modulates the expression of virulence factors and stress-response genes through RNAIII-mediated post-transcriptional regulation, contributing to bacterial adaptation and competitive fitness [79,80].

In the rumen, QS may participate in environmental sensing, intercellular communication, and functional adaptation of microbial communities under stress conditions. However, these roles remain largely inferential and require validation through in vivo studies integrating microbial ecology, signaling molecule dynamics, and host physiological responses. Such insights may provide a basis for future exploration of signaling-mediated approaches to modulate rumen function and improve animal health and productivity under stress conditions.

## 5. QS-Mediated Regulation of Rumen Microbial Ecology

Stress-induced alterations in QS signaling represent endogenous microbial responses to changes in the rumen environment, whereas QS modulation strategies involve deliberate external manipulation of microbial communication systems. Based on the regulatory role of QS in microbial collective behaviors, modulation of QS signaling is increasingly being considered a potential strategy for managing rumen microecology. Unlike traditional dietary manipulation or antibiotic intervention, QS regulation specifically targets microbial communication and can exert indirect effects on microbial functions at the community level. While theoretically promising, the extent to which QS-based strategies can modulate ruminal microbial ecosystems in a specific and sustained manner remains unclear due to the limited direct evidence currently available.

### 5.1. Signal Enhancement

Under stress conditions, QS activity in some functionally important microorganisms, such as fiber-degrading or metabolite-producing bacteria, may be disrupted, and moderate enhancement of QS signaling may help restore microbial coordination and functional homeostasis. Previous studies in non-ruminal microbial models have demonstrated that exogenous AI-2 supplementation can promote biofilm formation in various microbial systems. For instance, low concentrations of exogenous AI-2 enhanced biofilm formation in *Pseudomonas aeruginosa* [81]. In *Lactobacillus plantarum*, exogenous AI-2 supplementation increased exopolysaccharide production and suppressed polysaccharide hydrolysis, thereby enhancing biofilm formation [82]. These findings provide mechanistic insights into the potential role of AI-2-mediated regulation of microbial cooperation; however, direct evidence demonstrating that exogenous AI-2 supplementation can beneficially modulate rumen microbial function or improve stress resilience in vivo is currently lacking. Therefore, AI-2-based manipulation should be considered a potential future strategy.

### 5.2. Signal Inhibition

Unlike the signal enhancement strategy, signal suppression aims to interfere with QS-mediated behaviors that may negatively affect microbial community balance or host health by disrupting QS signal production, recognition, or transduction pathways. In the LuxS/AI-2 system, some signal analogues with similar structures can compete to interfere with the signal recognition process. For example, D-ribose is structurally similar to AI-2, and it can inhibit virulence gene expression and biofilm formation in certain pathogenic bacteria by interfering with AI-2 signal recognition [83,84]. Similarly, 4-hydroxy-2,5-dimethyl-3(2H)-furanone (HDMF), a naturally occurring furanone compound, competes with AHL for binding to LuxR-type receptors, thereby antagonizing AHL-mediated signal transduction [85,86]. However, these results were obtained from single-species studies. In the complex microbial ecosystem of the rumen, the actual regulatory effects of such compounds on target microbial communities may differ from single-bacterial experiments. Beyond synthetic or signal-mimicking compounds, several plant-derived bioactive compounds have also been identified as potential QS modulators. Phenolic compounds, flavonoids, and coumarins have been reported to interfere with QS processes in bacterial models by affecting AHL synthesis, disrupting signal–receptor interactions, or promoting signal degradation [87,88,89,90]. Feeding studies in ruminants further indicate that supplementation with these phytochemicals can alter ruminal microbial community composition and fermentation profiles, suggesting their potential roles in shaping rumen microbial ecology [91]. However, these effects are generally attributed to multiple biological activities, including antimicrobial activity, antioxidant effects, and altered substrate utilization, and whether they function primarily as QS modulators in the rumen remains to be established [92,93].

### 5.3. Evidence from Ruminant Studies

Several preliminary studies in ruminants suggest that QS-related interventions may influence rumen microbial characteristics and host stress responses under practical feeding conditions. For instance, dietary supplementation with 1000 mg/day of HDMF enhances the antioxidant capacity and increases the abundances of certain disease-resistant bacteria by promoting AI-2 signaling molecules and biofilm formation in Hu sheep [94]. This observation differs from previous reports of HDMF-mediated QS inhibition, possibly due to differences between single-strain in vitro models and the complex rumen microbial ecosystem. In contrast, supplementation with D-ribose at 300 mg/kg dry matter (DM) over an 80-day feeding trial exerted opposite effects, which could effectively inhibit rumen microbial LuxS/AI-2 QS in Hu sheep; this additive reduced ruminal AI-2 levels and serum cortisol, elevated total microbial density and nutrient digestibility, and simultaneously improved antioxidant status and growth performance of lambs [95]. Moreover, dietary resveratrol supplementation at 150 mg/kg DM alleviated heat stress in Hu sheep by enhancing antioxidant capacity, reducing cortisol, and increasing ruminal microbial density, biofilm formation, and microbial crude protein; notably, AI-2 concentrations did not differ between groups [96].

Collectively, these studies provide preliminary evidence for the potential involvement of QS-related regulation in rumen microbial adaptation; however, interpretation remains limited by several factors, including the use of a single sheep breed, relatively modest sample sizes, and the lack of dose–response evaluations. In addition, AI-2 quantification was primarily based on colorimetric assays with limited specificity, while AHL signals and QS gene expression dynamics were not assessed. Therefore, the observed benefits to host antioxidant status, stress responses, and growth performance likely reflect a combination of QS-related and QS-independent mechanisms, rather than being the consequence of QS modulation alone. Overall, these findings suggest that QS signaling may represent one of several microbial regulatory pathways associated with the effects of certain dietary additives on ruminal microbial functions and host stress responses under practical production conditions.

**Figure 2:**
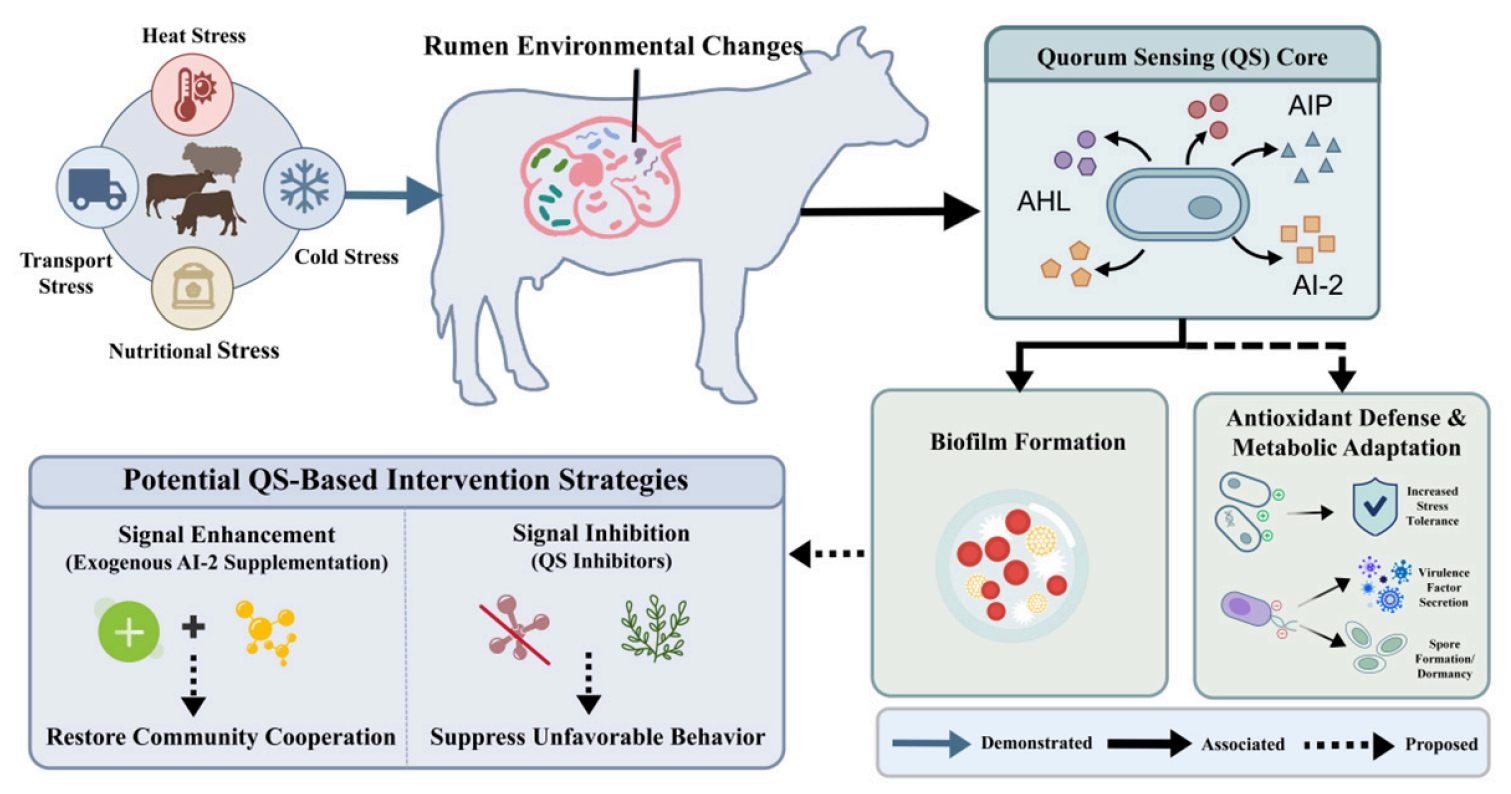
Quorum sensing-mediated microbial adaptation and potential intervention strategies in the rumen under environmental and nutritional stress. Solid blue arrows represent demonstrated mechanisms supported by experimental evidence, solid black arrows represent associated relationships based on indirect evidence, and dashed arrows represent proposed hypotheses requiring future validation. AI-2, autoinducer-2; AHL, acyl-homoserine lactones; AIP, autoinducer peptides.

In summary, based on evidence from microbial mechanisms and preliminary ruminant studies, we propose a conceptual framework linking environmental and nutritional stressors to shifts in the ruminal microenvironment, subsequent QS-mediated microbial functional adaptation, and the potential application of QS-targeted strategies to maintain rumen metabolic homeostasis (Figure 2).

## 6. Future Research Directions and Perspectives

Although the role of QS in microbial stress responses has attracted increasing attention in recent years, systematic investigations in the rumen, a highly complex anaerobic ecosystem, remain limited. Current studies have primarily focused on the detection of signaling molecules and their associations with fermentation functions, whereas the underlying regulatory mechanisms and community-level functional pathways remain poorly understood. To address these gaps and provide concrete methodological guidance, future research should prioritize three specific directions:

Establishment of standardized stress models. Because current studies on QS regulation are largely confined to in vitro systems or controlled experimental conditions, the responses and stability of QS systems within the highly dynamic rumen environment remain insufficiently understood. Future research should establish standardized in vivo stress models, including heat stress, cold stress, nutritional transition, and transport-related stress, to systematically evaluate stress-induced QS responses. Complementary in vitro fermentation systems, such as batch culture, continuous culture, and RUSITEC systems, should also be employed to dissect the specific effects of individual stressors and QS modulators under controlled conditions. These standardized platforms can improve comparability among studies by providing reproducible experimental conditions and allowing precise manipulation of key environmental factors, including pH, temperature, and substrate availability, thereby providing a foundation for investigating the ecological functions of QS in the rumen.

Continuous monitoring of QS dynamics. Based on these standardized models, future studies should focus on resolving the temporal characteristics of QS responses during stress exposure, adaptation, and recovery. Because most current studies rely on single time-point measurements, longitudinal sampling strategies and repeated measurements of QS signals are needed to capture dynamic changes in microbial communication. Targeted quantification of key QS molecules, including AI-2, AHL, and AIP, combined with continuous monitoring of rumen environmental parameters such as pH and temperature, will help clarify how environmental fluctuations regulate QS activity. Furthermore, the integration of metagenomics, metatranscriptomics, metabolomics, and microbial community profiling will facilitate the identification of QS-responsive microorganisms and functional pathways, and help determine whether QS contributes directly to microbial adaptation or reflects secondary ecological changes.

Validation of QS modulators. Building upon an improved understanding of QS responses under stress conditions, future research should further evaluate the practical potential of QS-based interventions. The stability, specificity, dose–response relationships, and long-term effects of different QS modulators require validation within the rumen environment through in vivo studies and subsequent evaluation under commercial production conditions. Such studies will be essential for determining whether QS-targeted approaches can be effectively incorporated into precision nutrition strategies. In the future, integration of QS-based interventions with biosensing technologies and precision nutrition may provide a conceptual basis for adaptive management approaches linking “stress detection–signal intervention–functional recovery”, thereby improving rumen resilience and supporting sustainable ruminant production.

## 7. Conclusions

Current evidence suggests that QS may contribute to rumen microbial adaptation under stress conditions by coordinating collective behavior. However, direct evidence supporting QS-mediated adaptation within the rumen remains limited, particularly under environmental challenges such as heat stress and cold stress. Most available insights are derived from nutritional interventions, in vitro studies, or non-ruminal microbial models, and the underlying ecological mechanisms within the complex rumen ecosystem remain largely uncharacterized. This review proposes a conceptual framework of “homeostatic change–signal response–collective behavior regulation” to illustrate how stress-induced environmental alterations may interact with microbial communication networks. Future studies integrating multi-omics approaches and functional validation are needed to clarify QS-mediated mechanisms and evaluate their potential for targeted regulation of rumen resilience and microbial stability.

## Figures and Tables

**Table 1:** Effects of different stressors on ruminal environment, microbial community, and QS.

Stress Type	Rumen Environmental Alterations	Microbial Community Shifts	QS Signaling Responses	Evidence Type	References
Heat stress	Reduced ruminal pH; weakened buffering capacity; altered substrate availability; increased thermal load	Increased abundance of lactate-producing and amylolytic bacteria; decreased abundance of fibrolytic bacteria	QS signaling molecule concentrations remained relatively stable under heat-stress alleviation conditions	Direct rumen QS evidence	[28,30,66]
Cold stress	Lower rumen temperature; changes in fermentation characteristics during winter	Microbial diversity increased; adaptive shifts in fiber-degrading bacteria	Increased drinking water temperature elevated AI-2 concentrations and enhanced biofilm formation	Direct rumen QS evidence	[38,67]
Transport stress	Fasting; dehydration; reduced ruminal pH; altered fermentation substrates	*Fibrobacter succinogenes* and *Ruminococcus flavefaciens* transiently increased (within 6 h, returning to baseline by day 15); soluble carbohydrate-utilizing bacteria increased	Direct evidence of QS changes is currently unavailable	Hypothetical/extrapolated mechanisms	[43,44]
Nutritional stress (Dietary shift)	Increased fermentable carbohydrate intake; decreased ruminal pH; altered volatile fatty acid profiles	Increased abundance of *Lactobacillus* and *Streptococcus* spp.; decreased abundance of *Fibrobacter* and *Ruminococcus* spp.	Increased AI-2 concentration; enhanced biofilm formation; upregulation of *ftsH* expression	Direct rumen QS evidence	[50,63]
Nutritional stress (Mycotoxin challenge)	Impaired fermentation; pH fluctuations; decreased substrate utilization	Altered microbial community structure with reduced abundance of key fibrolytic and methanogenic microorganisms	Reduced concentrations of AI-2 and multiple AHL signaling molecules	Indirect evidence from rumen simulation studies	[64,65]

Note: This table summarizes reported effects of different stressors on ruminal environmental parameters, microbial community composition, and QS signaling molecules based on previously published studies. Evidence type was categorized as direct rumen QS evidence, indirect evidence, or hypothetical/extrapolated mechanisms according to the level of experimental support. Changes in microbial taxa and QS-related molecules may vary depending on experimental conditions and analytical methods. QS, quorum sensing; AI-2, autoinducer-2; AHL, acyl-homoserine lactones.

**Table 2:** Summary of experimental models, stress conditions, analyzed QS molecules, and analytical methods in rumen-related QS studies.

Animal Species/Experimental Model	Stressor/Experimental Condition	QS Molecules	Analytical Methods	Main Findings	Reference
Hu sheep (in vivo feeding trial)	Nutritional stress: reduced dietary concentrate-to-forage ratio (from 75:25 to 49:51)	AI-2	Fe(III)-1,10-phenanthroline colorimetric assay	Lower concentrate proportion; elevated ruminal microbial density and AI-2 levels; promoted microbial biofilm formation; upregulated *ftsH* expression	[63]
RUSITEC system (in vitro)	Nutritional stress: zearalenone challenge	AI-2; C4-HSL	HPLC-FD (AI-2); UHPLC-MS/MS (C4-HSL)	Zearalenone exposure decreased AI-2 concentrations and exhibited a tendency to lower C4-HSL abundance, accompanied by impaired rumen function	[64]
RUSITEC system (in vitro)	Nutritional stress: aflatoxin B1 challenge	AI-2; C4-HSL; C6-HSL; 3-oxo-C6-HSL	HPLC-FD (AI-2); UHPLC-MS/MS (AHL)	Aflatoxin B1 suppressed the concentrations of all detected QS signals, disrupted ruminal bacterial community structure and weakened overall fermentation capacity	[65]
Cannulated Simmental beef bulls (in vivo feeding trial)	Heat stress: cooling intervention (mechanical ventilation and water spray)	AI-2; 3-oxo-C6-HSL	HPLC-FD (AI-2); UHPLC-MS (3-oxo-C6-HSL)	Heat stress and subsequent cooling reshaped ruminal microbiota and metabolome profiles, while the concentrations of AI-2 and 3-oxo-C6-HSL remained stable	[66]
Hu sheep (in vivo feeding trial)	Cold stress: increased drinking water temperature	AI-2	Fe(III)-1,10-phenanthroline colorimetric assay	Warm drinking water under cold stress increased ruminal AI-2 concentration, enhanced microbial biofilm formation and alleviated systemic oxidative stress	[67]

Note: QS, quorum sensing; AI-2, autoinducer-2; HPLC-FD, high-performance liquid chromatography with fluorescence detection; UHPLC-MS/MS, ultra-high-performance liquid chromatography-tandem mass spectrometry; RUSITEC, rumen simulation technique.

## Data Availability

No new data were created or analyzed in this study. Data sharing is not applicable.

## Abbreviations

The following abbreviations are used in this manuscript:

VFA	Volatile fatty acids
QS	Quorum sensing
AHL	N-acyl-homoserine lactones
AIP	Autoinducer peptides
AI-2	Autoinducer-2
HDMF	4-hydroxy-2,5-dimethyl-3(2H)-furanone
RUSITEC	Rumen simulation technique
DM	Dry matter
